# Effectiveness of mindfulness and Qigong training for self-healing in patients with Hwabyung and depressive disorder: a protocol for a randomized controlled trial

**DOI:** 10.3389/fpsyt.2024.1336656

**Published:** 2024-04-17

**Authors:** Seok-In Yoon, Hui-Yeong Park, Hyun Woo Lee, Chan Park, Sun-Yong Chung, Jong Woo Kim

**Affiliations:** ^1^Department of Neuropsychiatry, College of Korean Medicine, Kyung Hee University, Seoul, Republic of Korea; ^2^Department of Neuropsychiatry, Kyung Hee University Korean Medicine Hospital at Gangdong, Seoul, Republic of Korea

**Keywords:** Hwabyung, depressive disorder, mindfulness, Qigong, randomized controlled trial

## Abstract

**Background:**

Hwabyung is a Korean culture-bound syndrome characterized by anger-related physical and psychological symptoms. Depressive disorder is a common mental illness occurring worldwide, and has been reported to have a high comorbidity rate with Hwabyung. For patients with depressive disorders, differential diagnosis and combined treatment for Hwabyung should be considered. Mindfulness and Qigong may be effective alternatives for regulating emotions such as depression, anxiety, and anger. This study aims to investigate whether Mindfulness and Qigong Training for Self-Healing (MQT-SH) would improve emotional problems in patients with Hwabyung and depressive disorder.

**Methods:**

This study will be a two-arm block randomized controlled trial. A total of 64 participants will be recruited and randomly assigned to either experimental or control group. While the experimental group will perform MQT-SH for the first six weeks, the control group will receive no treatment. During the next six weeks, the control group will perform MQT-SH for ethical equity. Assessments will be conducted at baseline, post-intervention (6 weeks) and follow-up (12 weeks). The primary outcomes would be Hwabyung and depression, while the secondary outcomes would include anxiety, anger, and vitality.

**Discussion:**

This study will provide a basis for assessing the effectiveness of mindfulness and Qigong training in patients with Hwabyung and depressive disorder.

**Ethics and dissemination:**

This study was approved by the Institutional Review Board of Kyung-Hee University Oriental Medicine Hospital in Gangdong (KHNMCOH 2023-09-003). The results will be disseminated through peer-reviewed publications.

**Trial registration:**

This trial was registered with the Clinical Research Information Service (CRIS), Republic of Korea, No. KCT0008937 and was registered on November 10, 2023.

## 1 Introduction

Hwabyung is a disorder that is culturally specific to Korea (1). It is a syndrome in which negative emotions such as anger cannot be resolved and explode. According to previous research, physical symptoms include chest stuffiness, heat, feeling of something pushing up, and the feeling of a lump in the throat or pit of the stomach (2). Psychological symptoms include feelings of unfairness, resentment, and anger (2). Symptoms have been reported to be associated with specific stressful events (3). Research on the diagnosis of Hwabyung commenced in the 2000s, and the diagnostic criteria were first established by Kim et al. (2). When standardized diagnostic criteria are applied, the prevalence of Hwabyung in Koreans has been reported to range between 4.2-13.3% (4–7). Treatment for Hwabyung is generally provided by Korean medicine doctors (3).

In Korean medicine, it is believed that Hwabyung manifests as a result of problems with the circulation and regulation of ‘Qi.’ According to Donguibogam, a classic in Korean medicine literature, physical and psychological problems occur when Qi is not properly circulated (通卽不痛 不通卽痛) (8). Hwabyung is understood as a state of psychological blockage caused by persistent unresolved resentment in which the emotion of anger is biased upward and, as a consequence, Qi cannot circulate properly (9). To treat these symptoms, Korean medicine aims to achieve balance and harmony between the mind and body through the optimal circulation of Qi.

Depression is a mental health problem characterized by absence of positive emotions such as interest and pleasure, and a depressed mood (10). It is a mental illness that not only negatively affects an individual's quality of life, but also their overall occupational and social functioning (11). Depressive disorder is a common disease occurring worldwide. More than 185 million people were diagnosed with depression in 2019, and its prevalence is reportedly higher among women (3.07%) than men (1.89%) (12). In the 2019 Global Burden of Disease Study, the disease burden of depressive disorder ranked 13th among the top 25 diseases, showing a disease burden similar to that of AIDS, cirrhosis, lung cancer, and headache (13). In particular, it can be said that symptoms of depression have become more serious due to the recent impact of COVID-19 (14).

Hwabyung and depressive disorder have a high comorbidity rate. Epidemiological surveys have shown that 44% of the patients with Hwabyung have depressive disorder (15). Considering the fact that previous research (16) has demonstrated that 28.5% of patients with depressive disorder have generalized anxiety disorder as a comorbidity, it can be considered that Hwabyung and depressive disorder have a very high comorbidity rate. Therefore, in patients with depressive disorder, differential diagnosis and combined treatment for Hwabyung should be considered.

Recently, various mind-body interventions, such as mindfulness and Qigong, have been proposed as alternatives for treating mental disorders while minimizing the side effects of drugs. Mindfulness, which based on traditional Buddhist practices, is operationally defined as “paying attention in a particular way: on purpose, in the present moment, and nonjudgmentally” (17). Mindfulness, a non-judgmental concentration on the present moment, results in a relaxation response associated with activation of the parasympathetic nervous system (18). Mindfulness helps patients with depression or Hwabyung stay in the present moment and not trapped in negative thoughts or past resentment (19, 20). Based on these principles of mindfulness, a standardized program has been developed for emotional regulation, including stress reduction and prevention of depressive relapse (19, 21). In fact, mindfulness-based programs have been shown to improve depression in patients with depressive disorders and university students (22, 23), as well as Hwabyung in middle-aged females (20).

Qigong, which has its basis in the Taoist tradition, is a system of practices intended to cultivate control over life energy, known as Qi, through various body postures and movements, deep breathing, and mental concentration (24). Qigong is a broad concept that includes both static and movement-based practices, and is classified into various methods depending on tradition, such as Ba Duan Jin, Liu Zi Jue, Wu Qin Xi, and Kouksundo (25). The broad concept of Qigong and its diverse methods make it difficult to standardize programs. To compensate for these limitations and increase reproducibility in follow-up studies, this study defined Qigong operationally as feeling, accumulating, and utilizing Qi. Previous studies have shown that Qigong improves depression and Hwabyung (26, 27). The effects of Qigong on emotional regulation are explained by mindfulness. Qigong, along with Tai Chi and yoga, is referred to as meditative or mindful movement (28, 29), and is considered to share the therapeutic mechanisms of mindfulness meditation for emotional regulation (30). From a Korean medicine perspective, Qigong is believed to ameliorate Hwabyung and depression through the regulation of Qi (8, 9).

This study aims to investigate the effectiveness of Mindfulness and Qigong Training for Self-Healing (MQT-SH) from the comorbidity of depression and Hwabyung. The MQT-SH is a standardized Korean medicine mind-body intervention that aims to achieve a healthy balance between the mind and body (9). The primary hypothesis is that MQT-SH significantly reduces Hwabyung and depression in comparison to the control group. The secondary hypothesis is that MQT-SH reduces anxiety and anger while increasing vitality compared to the control group.

## 2 Methods

### 2.1 Trial design

This study is designed as a two-arm block randomized controlled trial. Patients will be assigned to either the experimental or the control group. The trial will be conducted for a duration of 12 weeks. During the first six weeks, the experimental group will receive MQT-SH, while the control group will receive no treatment as a waiting list control (WLC). During the next six weeks, the control group will receive MQT-SH with consideration for ethical equity. Assessments will be performed at three points: baseline (T1), post-intervention (T2; 6 weeks), and follow-up (T3; 12 weeks).

**Figure 1:**
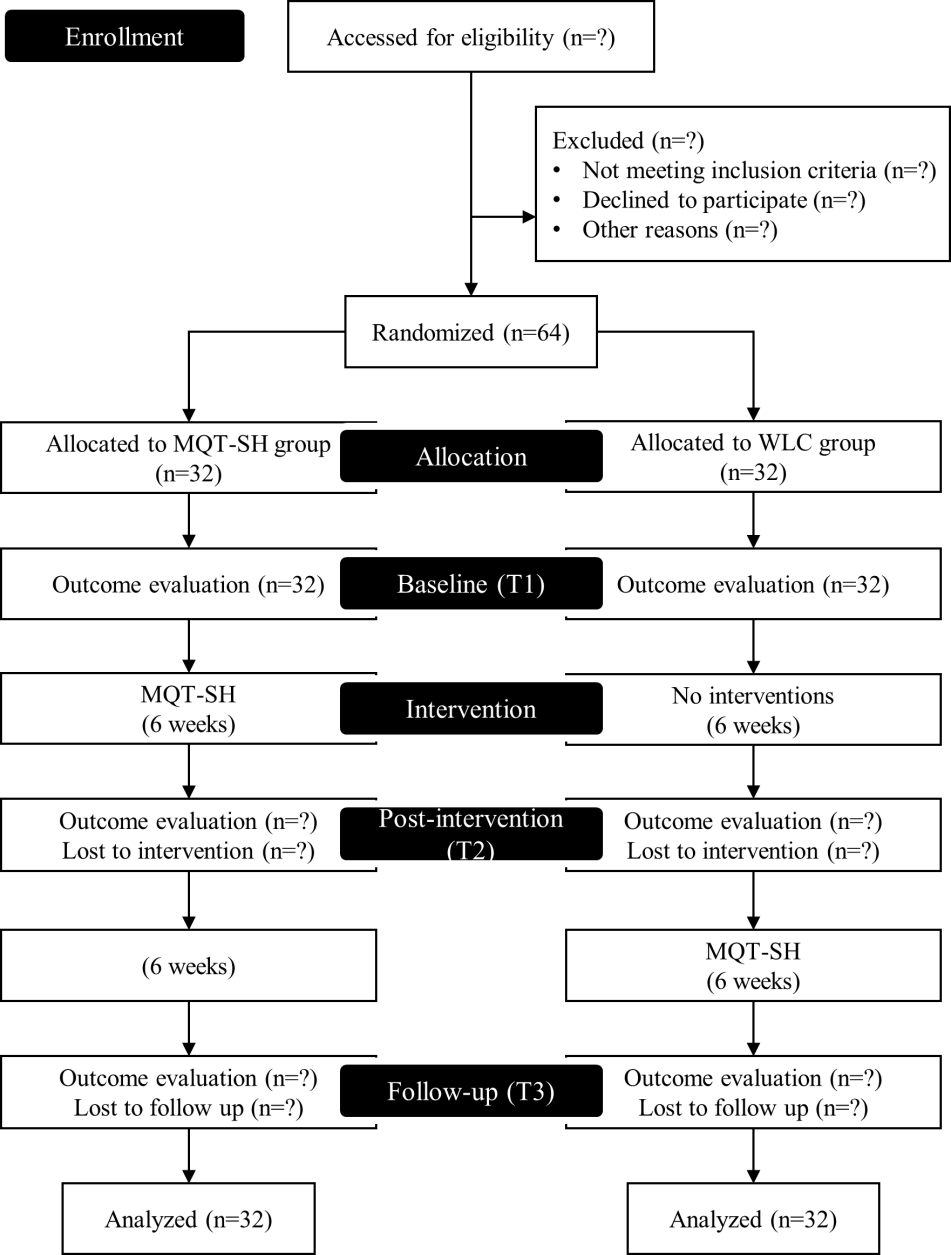
Study flow chart.

This trial was registered with the Clinical Research Information Service (CRIS), Republic of Korea (KCT0008937). This trial complies with the SPIRIT guidelines and will be conducted in accordance with the tenets of Declaration of Helsinki. **Figure 1** presents a flow chart representing the overview of the study design.

### 2.2 Study setting

This trial will be conducted at Kyung Hee University Korean Medicine Hospital in Gangdong, South Korea. Participants will be recruited through advertisements in hospital and subways. Potential participants interested in participating will be informed about the study. If they agree to participate, they will be screened to assess the inclusion and exclusion criteria.

### 2.3 Eligibility criteria

Participants will be included if they i) have simultaneously been diagnosed with Hwabyung and depressive disorder by the Hwa-Byung Diagnostic Interview Schedule (HBDIS) (2) and Structured Clinical Interview for DSM-5 (SCID-5) (31); ii) are ≥ 19 years of age; and iii) can participate in the study by considering the timetable.

### 2.4 Interventions

Participants will be randomly assigned to either MQT-SH or WLC.

#### 2.4.1 Mindfulness and Qigong training for self-healing

The experimental group will perform MQT-SH for the first six weeks. MQT-SH is a standardized mindfulness- and Qigong-based program to enhance self-healing (9). The goal of MQT-SH is to achieve optimal physical and psychological conditions. To achieve this goal, MQT-SH consists of two modules: i) balance and harmony, and ii) circulation and interchange. According to the traditional Oriental medicine, excessive emotions cause physical and psychological problems (32), and these imbalanced problems can be solved through circulation and interchange of energy (33). Balance and harmony have recently become the core principles of health and well-being across various cultures (34–36).

The balance and harmony module consists of the 1st to 3rd sessions. This module emphasizes the balance of inhalation/exhalation, sympathetic/parasympathetic nervous systems, and past/future/present for health. This module teaches various mindfulness-based training for healthy balance. In the first session, the participants will be taught the meaning of breathing and how to use it. Subsequently, breathing meditation will be performed. Breathing meditation involves counting numbers or adding words while concentrating on breathing. In the second session, participants will be educated about the balance and harmony of stress and relaxation. After educating the patients, body scan and autogenic training will be performed. In the third session, the participants would be educated on the meaning of mindfulness, and sitting meditation will be performed subsequently. Sitting meditation focuses on the awareness of breathing and bodily sensations.

The circulation and interchange module consists of the 4th to 6th sessions. This module teaches Qigong to encourage proper circulation and interchange of Qi. In MQT-SH, Qigong is operationally defined in three ways: feeling, accumulating, and utilizing Qi. In the fourth session, participants will be educated about the energy that could be experienced phenomenologically, and after education, meditation with Qi will be performed. The goal of meditating with Qi is to sensuously experience Qi in a comfortable and calm state. Meditation with Qi consists of breathing meditation, body scan, and mindfulness of the energy called Qi. In the fifth session, two ways of obtaining energy (e.g., breathing and eating) would be taught to the participants. Moreover, Danjeon breathing and eating meditations will be performed. In the sixth session, participants will be taught to use energy, and simple Qigong movements and walking meditation will be performed. **Table 1** provides the detailed structure by session.

**Table 1:** Sessions of the intervention.

Module	Session	Subject	Education (20 min)	Practice (40 min)	Group activities (30 min)
Balance and harmony	1	Breathing	Understanding breathing; How to use breathing	Breathing meditation	1) Sharing experiences 2) Question and answer
	2	Relaxation	Balance and harmony of stress and relaxation	Autogenic training; Body scan	1) Checking home training 2) Sharing experiences 3) Question and answer
	3	Mindfulness	Understanding mindfulness	Sitting meditation (breathing and bodily sensations)	
Circulation and interchange	4	Feeling Qi	Energy that can be experienced phenomenologically	Meditation with Qi 1) Breathing meditation 2) Body scan 3) Mindfulness of Qi	1) Checking home training 2) Sharing experiences 3) Question and answer
	5	Accumulating Qi	Two ways to obtain energy (e.g. breathing and eating)	Danjeon breathing; Eating meditation	
	6	Utilizing Qi	How to use energy	Qigong movements; Walking meditation	

The MQT-SH period may vary depending on the setting (9). A manual for researchers and participants was developed to improve compliance to the intervention protocol. In this study, the MQT-SH will be conducted once a week for 1.5 h for a total of six weeks. Each session will consist of 20 min of education, 40 min of practice (i.e., meditation), and 30 min of group activities, such as checking home training and sharing practice experiences. This intervention will be conducted as a group program, with a maximum of 12 people per group. Participants will be asked to perform home training five times a week for at least 30 min each day. Participants will receive guided audio materials for home training. Dropouts will be defined as i) individuals attending three or less out of six sessions, ii) those who complain of physical or psychological discomfort and want to give up because of such discomfort, or iii) those who would want to give up for reasons other than discomfort.

#### 2.4.2 Wait-list control

The control group will be assigned to WLC and will receive no treatment for the first six weeks. Considering ethical equity, the control group will undergo the same MQT-SH over the next six weeks.

### 2.5 Sample size

The sample size was estimated using G*power (37). The sample size calculation is based on a power of 80% and a type I error of 5%, as well as a moderate effect size for the intervention (partial *η*^2^ = 0.04). A minimum sample size of 25 participants per group is estimated (50 participants in total). This number was obtained by performing a simulation procedure using repeated-measures ANOVA, considering the existence of a significant 2 (group: experimental *vs*. control) × 2 (time: T1 *vs*. T2) interaction effect. Considering a dropout rate of 20%, we aim to include 32 participants per group (64 participants in total).

### 2.6 Randomization

Participants will be randomly allocated to either the experimental or the control group (1:1 ratio, block size = 4). Random numbers for allocation will be generated by the computer and managed by the researcher (YSI). Before enrollment, the participants and other researchers will be unaware of the allocation. After enrollment, YSI will allocate participants to the experimental or control group according to random numbers, and the results of the allocation will be shared only with the clinical research coordinator.

### 2.7 Blinding

In this clinical trial, assessors will be blinded. However, participants and practitioners will not be blinded. The assessors will be blinded to the intervention group and prevented from speaking with the participants, except during the clinical interviews.

### 2.8 Assessment

Assessments are scheduled at baseline (T1), post-intervention (six weeks; T2), and follow-up (12 weeks; T3). Assessments include observer ratings (conducted by blinded clinicians) and self-report ratings. A structured interview manual will be used to increase the reliability of observer ratings (e.g., 38, 39). If participants drop out, they will not be assessed further. Missing data will be statistically imputed. **Table 2** summarizes the assessment time points.

**Table 2:** Study assessment points.

	Screening	T1	T2	T3
HBDIS	○			
SCID-5	○			
Demographic Information	○			
HS		○	○	○
HCT		○	○	○
HDRS		○	○	○
HARS		○	○	○
STAXI		○	○	○
IVS		○	○	○

T1, baseline; T2, post-intervention; T3, follow-up; HBDIS, Hwa-Byung Diagnostic Interview Schedule; SCID-5, Structured Clinical Interview for DSM-5; HS, Hwabyung Scale; HCT, Hwabyung Comprehensive Test; HDRS, Hamilton Depression Rating Scale; HARS, Hamilton Anxiety Rating Scale; STAXI; State-Trait Anger Expression Inventory; IVS, Integrative Vitality Scale.

### 2.9 Primary outcomes

#### 2.9.1 Hwabyung scale

The Hwabyung scale (40) will be used to assess Hwabyung-related personality and symptoms. This scale comprises 16 items on Hwabyung personality and 15 items on Hwabyung symptoms. This is a self-reported 5-point Likert scale. The total score ranges from 0 to 64 for Hwabyung personality, and from 0 to 60 for Hwabyung symptoms. Higher scores indicate higher Hwabyung personality and symptoms. In a previous study (40), the Cronbach's alpha for the Hwabyung scale was .85 for Hwabyung personality, .93 for Hwabyung symptoms, and .93 overall.

#### 2.9.2 Hwabyung comprehensive test

The Hwabyung Comprehensive Test (41) will be used to assess Hwabyung-related symptoms and psychological characteristics. This test consists of 13 items on Hwabyung symptoms, 5 items on the incident questionnaire, and 21 items on Hwabyung psychological characteristics. Hwabyung symptoms and psychological characteristics will be used to interpret scores. This test is a self-reported 5-point Likert scale. The total score ranges from 0 to 52 for Hwabyung symptoms, and from 0 to 84 for Hwabyung psychological characteristics. Higher score indicates more severe Hwabyung symptoms and vulnerability. In a previous study (41), the Cronbach's alpha for the Hwabyung Comprehensive Test was .89 for Hwabyung symptoms and .95 for Hwabyung psychological characteristics.

#### 2.9.3 Hamilton depression rating scale

In this study, a structured interview manual will be used to increase inter-rater reliability.

The Korean version of the Hamilton Depression Rating Scale (42) will be used to assess depression. This is an observer rating scale consisting of 17 items. Each item is scored from 0 to 2 or from 0 to 4. The total score ranges from 0 to 52. Higher scores indicate more depressive symptoms. In this study, a structured interview manual will be used to increase the inter-rater reliability (39).

### 2.10 Secondary outcomes

#### 2.10.1 Hamilton anxiety rating scale

The Hamilton Anxiety Rating Scale (43) will be used to assess anxiety. This is an observer rating scale consisting of 14 items. Each item is scored from 0 to 4. The total score ranges from 0 to 56. Higher scores indicate higher anxiety symptoms. In this study, a structured interview manual will be used to increase the inter-rater reliability (38).

#### 2.10.2 State-trait anger expression inventory

The Korean Adaptation of the State-Trait Anger Expression Inventory (44) will be used to assess anger experiences and expression. This scale consists of 10 items for state anger, 10 items for trait anger, and 24 items for anger expression. This is a self-reported 4-point Likert scale. The total score ranges from 10 to 40 for state anger, 10 to 40 for trait anger, and 24 to 96 for anger expression. Anger expression consists of three factors: anger-out, anger-in, and anger control. Anger control is considered a functional expression of anger and will be reverse calculated. A higher score indicates experiencing and expressing more anger. In a previous study (44), Cronbach's alpha was .90 for state anger, .75 for trait anger, .70 for anger-out, .66 for anger-in and, .79 for anger-control.

#### 2.10.3 Integrative vitality scale

The Integrative Vitality Scale is a self-reported questionnaire that is under development to measure physical and psychological vitality. This scale consists of 11 items on physical vitality (e.g., My body is full of energy) and 11 items on psychological vitality (e.g., I have a passion for life). This is a self-reported 5-point Likert scale. The total score ranges from 0 to 44 for physical vitality and from 0 to 44 for psychological vitality. One item (e.g., My head feels heavy and achy) will be reverse-calculated. Higher scores indicate higher subjective vitality. In a survey of 348 people, Cronbach's alpha was .91 for physical vitality, .91 for psychological vitality, and .94 overall (preparing for publication).

### 2.11 Adverse events

Adverse events will be assessed after each session of MQT-SH. The results section will describe the type, frequency, and severity of the adverse events. Severity will be assessed according to three grades (mild, moderate, or severe). Mild is defined as an adverse reaction that does not interfere with daily life. Moderate is defined as an adverse reaction that interferes with daily life but is not dangerous. Severe is defined as an adverse reaction that is severe and interferes with basic daily activities (e.g., eating, changing clothes).

### 2.12 Data management

All the data will be encrypted and stored on the researcher's computer. Personally identifiable information will not be included in publications. Personal information will be stored together with the data for a maximum of 10 years, after which they will be destroyed.

### 2.13 Statistical analyses

Analyses based on the Intention-To-Treat (ITT) principle will be conducted, with missing data imputed using expectation-maximization (EM) algorithm.

To investigate the effect of MQT-SH, 2 (group: MQT-SH *vs*. WLC) × 2 (time: T1 *vs*. T2) repeated measures ANOVA will be performed. If the interaction between the group and time is significant, a simple main effect analysis will be performed.

Additional analyses will be performed. First, to investigate the follow-up effect of the MQT-SH, a paired t-test will be performed to compare T3 and T1 and T3 and T2 in the experimental group alone. Second, a paired t-test comparing T3 and T2 in the control group will be performed to investigate the effects of MQT-SH. Third, a subgroup analysis will be performed to investigate the difference in effectiveness depending on the amount of home training. For this purpose, 2 (amount: top 30% *vs*. bottom 30%) × 2 (time: pre-intervention *vs*. post-intervention) repeated-measures ANOVA will be performed. Data from both the experimental and control groups will be used for subgroup analysis.

## 3 Discussion

The purpose of this study is to investigate the effectiveness of a mindfulness and Qigong program for patients with depression and Hwabyung. This study is designed as a two-armed block randomized controlled trial. This study will primarily investigate the effectiveness of MQT-SH on Hwabyung, depression, anxiety, anger, and vitality. Additionally, this study will investigate the follow-up effect of MQT-SH and perform a subgroup analysis to compare the differences in the effect according to the amount of home training.

While depression is characterized by a sad mood or absence of joy or interest (10), Hwabyung is characterized by a combination of physical (chest tightness, heat, etc.) and psychological (resentment, resentment, etc.) symptoms related to anger (2). Although depression and Hwabyung have different diagnostic criteria, they have a high likelihood of comorbidity (15). In fact, 44.1% of patients with Hwabyung were diagnosed with depressive disorder, and 18.3% were diagnosed with both depressive and anxiety disorders (45). This suggests that Hwabyung is not only related to depression, but also to anxiety. Therefore, interventions are needed to comprehensively deal with various emotional problems such as depression, anxiety, and anger.

Mindfulness and Qigong can be used as transdiagnostic approaches for improving emotion regulation. Previous studies have shown that mind-body interventions, such as mindfulness and Qigong, improve depression, anxiety, and anger (27, 46–48). Mindfulness is a basic factor in several mind-body interventions and is considered a Qigong therapeutic mechanism for emotional regulation (20). This study investigates whether mindfulness and Qigong interventions improve emotional regulation in patients with Hwabyung and depressive disorder. MQT-SH is a standardized mind-body intervention that combines mindfulness and Qigong (9). This study adhered to the standard procedure of MQT-SH, but made some modifications to the period and content in consideration of the study setting and participants.

The control group in this study is a non-active group. For ethical reasons, the control group will perform MQT-SH for six weeks between the T2 and T3 assessments. Therefore, the main statistical analysis is a comparison of the two groups for T1 and T2 assessments. Additionally, a follow-up effect analysis will be conducted in the experimental group alone, and a single-arm pre-post effect analysis will be conducted in the control group. Finally, a subgroup analysis will be conducted based on the amount of home training.

### 3.1 Limitations

The results of this study may have several limitations. First, the participants and intervention instructor are not blinded. The control group is a non-active group; therefore, the participants are not blinded. In addition, due to the nature of the intervention, where meditation and Qigong are directly guided, the intervention instructor also cannot be blinded. However, the researchers conducting observer ratings will be blinded. Second, this study is a single-center trial. A single-center approach has the advantage of complying with the study protocol in comparison to a multicenter trial. However, this may have implications for generalizability and external validity.

### 3.2 Conclusion

In conclusion, this study will provide empirical results on the effectiveness of a standardized mindfulness and Qigong program for patients with Hwabyung and depressive disorder. This study will be an opportunity to investigate the possibility of a low-risk practical treatment for emotional problems in clinical settings.

### 3.3 Trial status

Recruitment, enrollment and treatment of participants has started. Follow-up assessments for the last participant are expected to be completed by September 2024.

## Ethics statement

This study was approved by the Institutional Review Board (IRB) of Kyung-Hee University Oriental Medicine Hospital in Gangdong (KHNMCOH 2023-09-003). Written informed consent will be obtained from all the participants prior to their inclusion in the study. If the study protocol is changed, it will be reviewed by the IRB. The results of this study will be disseminated through publication submissions in peer-reviewed journals.

## Author contributions

S-IY: Conceptualization, Writing – original draft. H-YP: Writing – original draft. HL: Writing – review & editing. CP: Writing – review & editing. S-YC: Conceptualization, Writing – review & editing. JWK: Conceptualization, Funding acquisition, Writing – review & editing.
